# G protein-coupled receptors: computer-aided ligand discovery and computational structural analyses in the 2010s

**DOI:** 10.1186/2193-9616-1-20

**Published:** 2013-12-20

**Authors:** Stefano Costanzi

**Affiliations:** Department of Chemistry and Center for Behavioral Neuroscience, American University, 4400 Massachusetts Ave, NW, 20016 Washington, DC USA

**Keywords:** G protein-coupled receptors, GPCRs, Camerino symposium, Water molecules, Homology modeling, Molecular docking, Graph theory

## Abstract

G protein-coupled receptors, or GPCRs, are a large superfamily of proteins found on the plasma membrane of cells. They are involved in most physiological and pathophysiological functions and constitute the target of the majority of marketed drugs. Although these receptors have been historically elusive to attempts of structural determination, GPCR crystallography is now in full blossom, opening the way to structure-based drug discovery and enabling homology modeling. This thematic issue of the journal *In Silico Pharmacology*, which illustrates how the expanding body of structural knowledge is fostering complex computational analyses of the structure-function relationships of the receptors and their interactions with their ligands, stems from the 31st Camerino-Cyprus-Noordwijkerhout Symposium held in Italy, in May 2013, at the University of Camerino. Specifically, it originates from a session of the symposium entitled “Structure-Based Discovery of Ligands of G Protein-Coupled Receptors: Finally a Reality”, and features a mix of research articles and reviews on the application of computational modeling to the analysis of the structure of GPCRs and the interactions of the receptors with their ligands.

G protein-coupled receptors, or GPCRs, are a large superfamily of proteins found on the plasma membrane of cells, i.e. the membrane that provides the border between the interior of a cell and the extracellular milieu (Pierce et al. 2002). They are the object of the research of the 2012 winners of the Nobel Prize in Chemistry Robert Lefkowitz and Brian Kobilka, who, with their seminal studies, gave a remarkable contribution to the advancement of the body of knowledge surrounding these receptor proteins.

Being located on the plasma membrane, GPCRs are essential components of the mechanism that allows cells to receive signals from their environment and react to them and are involved in most physiological and pathophysiological functions. Hence, they constitute the target of the majority of the targeted drugs. The biological response consequent the interaction of GPCRs with natural ligands or drugs arises from the simultaneous modulation of a variety of signaling pathways. Some of these pathways are mediated by the coupling of the receptors with intracellular heterotrimeric guanine nucleotide binding proteins known as G proteins, hence the name of GPCRs. Moreover, studies pioneered by Robert Lefkowitz demonstrated that alternative GPCR signaling pathways are mediated by the activation of proteins known as arrestins (Pierce et al. 2002; Wisler et al. 2007; Kahsai et al. 2011; Lefkowitz & Shenoy 2005; Martin et al. 2004; Reiter et al. 2012; Rajagopal et al. 2011; Drake et al. 2008; Lefkowitz 2013; Kobilka 2013).

Historically elusive to attempts of structural determination, GPCRs conceded for the first time to the efforts of crystallographers in the year 2000, when Palczewski and coworkers solved for the first time the three-dimentional structure of rhodopsin (Palczewski et al. 2000). Rhodopsin is a peculiar GPCR that, unlike most members of the superfamily, is not activated by diffusible ligands. Conversely, it is a photoreceptor naturally activated by light, which triggers the isomerization of a covalently bound inverse agonist, i.e. a molecule that suppresses the activity of the receptor, into an agonist, i.e. a molecule that activates the receptor (Costanzi et al. 2009). Rhodopsin remained the only GPCR with experimentally elucidated structures until 2007, when the first structures of the β_2_ adrenergic receptor were solved (Cherezov et al. 2007; Rasmussen et al. 2007; Rosenbaum et al. 2007). Thanks to the introduction of a number of expedients, which include the use of antibodies, fusion proteins, stabilizing mutations and stabilizing ligands, GPCR crystallography is now in full blossom (Stevens et al. 2013; Katritch et al. 2013; Venkatakrishnan et al. 2013; Tate & Schertler 2009; Kruse et al. 2013; Steyaert & Kobilka 2011). At the time of this writing, over 80 structures for over 20 distinct receptors have been solved.

The GPCR structures that are now available are paving the way for structure-based drug discovery, i.e. the rational identification of novel active molecules based on computer-aided analyses of their interactions with their target receptor (Mason et al. 2012; Congreve et al. 2011; Congreve & Marshall 2009; Jacobson & Costanzi 2012; Lane et al. 2013). Moreover, the GPCRs for which crystal structures have been solved provide templates for the construction of models for the remaining members of the superfamily through a technique know as homology modeling and based on the observation that the structures of evolutionarily related proteins, such as GPCRs are, are closely related to each other (Costanzi & Wang 2014; Costanzi 2013; Costanzi 2010; Costanzi 2008).

This thematic issue of the journal *In Silico Pharmacology*, which illustrates how the expanding body of structural knowledge is fostering complex computational analyses of the structure-function relationships of the receptors and their interactions with their ligands, stems from the 31st Camerino-Cyprus-Noordwijkerhout Symposium held in Italy, in May 2013, at the University of Camerino. Specifically, it originates from a session of the symposium entitled “Structure-Based Discovery of Ligands of G Protein-Coupled Receptors: Finally a Reality”. The issue is opened by an article from Giannella and Angeli, who provide an insightful overview of the evolution of the field of GPCR studies observed from a very special vantage point: the international symposia that are regularly held in Camerino since the late 1970s (Giannella & Angeli 2013). The opening piece is followed by three articles that discuss the implications of the recent advancements in GPCR crystallography for computer-aided ligand discovery: a commentary from Jacobson that illustrates the impact of the solution of GPCR structures on medicinal chemistry efforts for the identification and the development of modulators of pharmaceutically relevant receptors (Jacobson 2013); an article from Mason, Marshall and coworkers that demonstrates how the rational computer-assisted design of GPCR ligands is finally enabled by the availability of GPCR structures as well as the development of techniques that account for the interaction of small molecules with the networks of water molecules and lipophilic hotspots that characterize their target receptors (Mason et al. 2013); an article from Dal Ben, Volpini and coworkers that illustrates how molecular docking targeting GPCR homology models derived from the crystal structures of closely receptors can be applied to the rationalization of structure-activity relationships, thus setting the stage for drug design (Dal Ben et al. 2013). The special issue is closed by two articles that describe computational analyses enabled by the availability of GPCR structures. The first one is an article from Floris, Moro and coworkers, which describes the development of a tool that, given a ligand of interest, facilitates the selection of the most suitable crystal structure for the study of the interactions of the crystallized receptor with that ligand or for the construction of a homology model of different receptor and the study of its interactions with that ligand (Floris et al. 2013). The authors implemented the tool in “Adenosiland”, a web-based platform dedicated to GPCRs activated by the nucleoside adenosine. The second one is an article from Sheftel, Costanzi and coworkers, which describes how the structure of GPCRs can be analyzed through graph theory techniques to highlight their structural features (Sheftel et al. 2013).
